# Ascending Palatine Artery Embolization for the Treatment of Surgically Refractory Post-tonsillectomy Hemorrhage

**DOI:** 10.7759/cureus.35985

**Published:** 2023-03-10

**Authors:** Ashish Kulhari

**Affiliations:** 1 Neurology, University of Missouri Kansas City School of Medicine, Kansas City, USA; 2 Medicine/Neurology, Kansas City University of Medicine and Biosciences, Kansas City, USA; 3 Neurology, Research Medical Center, Kansas City, USA

**Keywords:** tonsillectomy, endovascular procedures, interventional radiology, endovascular coil embolization, post-tonsillectomy bleeding

## Abstract

Severe uncontrollable surgically refractory postoperative bleeding after tonsillectomy is a rare but potentially life-threatening event. The efficacy of surgical interventions tends to decline with repetition, which leads to higher morbidity and mortality. Endovascular embolization of the external carotid artery branches as an effective treatment in surgically refractory post-tonsillectomy hemorrhage has been described previously. We describe the case of a 27-year-old man who presented with surgically refractory post-tonsillectomy hemorrhage and underwent successful endovascular embolization of the ascending palatine artery with immediate hemostasis.

## Introduction

Tonsillectomy is among the most common surgical procedures in the United States [1]. Due to highly vascular anatomy, post-tonsillectomy hemorrhage remains the most common serious complication during or after surgery, with a reported incidence of around 3% [1,2]. Depending on the severity of the post-tonsillectomy hemorrhage, initial management is usually observation or surgical intervention. The efficacy of surgical interventions tends to decline with repetition, which leads to higher morbidity and mortality [2]. Evidence for endovascular embolization of the branches of the external carotid artery as an effective treatment for severe surgically refractory post-tonsillectomy hemorrhage has been growing [2-5]. We describe the case of a 27-year-old man who presented with surgically refractory post-tonsillectomy hemorrhage and underwent successful endovascular embolization of the ascending palatine artery with immediate hemostasis.

## Case presentation

Case

A 27-year-old man with no prior medical history underwent elective right-sided tonsillectomy for frequent tonsillar abscesses. He was discharged home the same day. On postoperative Day 4, he presented to the emergency room with severe oral bleeding from the surgical site. He was taken to the operating room emergently where a small arterial bleeder was noted in the right superior tonsillar fossa. The arterial bleeding site was cauterized with suction and bipolar cautery and the patient was discharged home. The patient returned to the emergency room within 24 hours with continued severe bleeding. The patient was taken again to the operating room where active bleeding was noted from the right mid and superior tonsillar pole. The bleeding site was cauterized with suction cautery and Surgicel (Ethicon Inc., Raritan, NJ) was placed in the tonsillar bed. Suturing of the tonsillar poles was attempted but could not be performed due to a lack of adequate tissue. Post-surgery, the patient continued to have bleeding from the surgical site. He was kept intubated and was transferred to our hospital for consideration of endovascular embolization. The patient was hemodynamically stable. Hemoglobin on admission to our hospital was 10.6 g/dl. The patient underwent diagnostic angiography of the right external carotid artery. The right tonsillar fossa blood supply was noted to be mainly from the right ascending (dominant) and descending palatine arteries. Superselective angiography of the right ascending and descending palatine arteries was performed. No obvious source of bleeding was identified. The right ascending palatine artery was embolized using coils. Oral packing was removed after the procedure. No obvious signs of bleeding were noted. The patient was successfully extubated on postoperative day 1 and discharged home on postoperative Day 3.

Procedure

**Figure 1:**
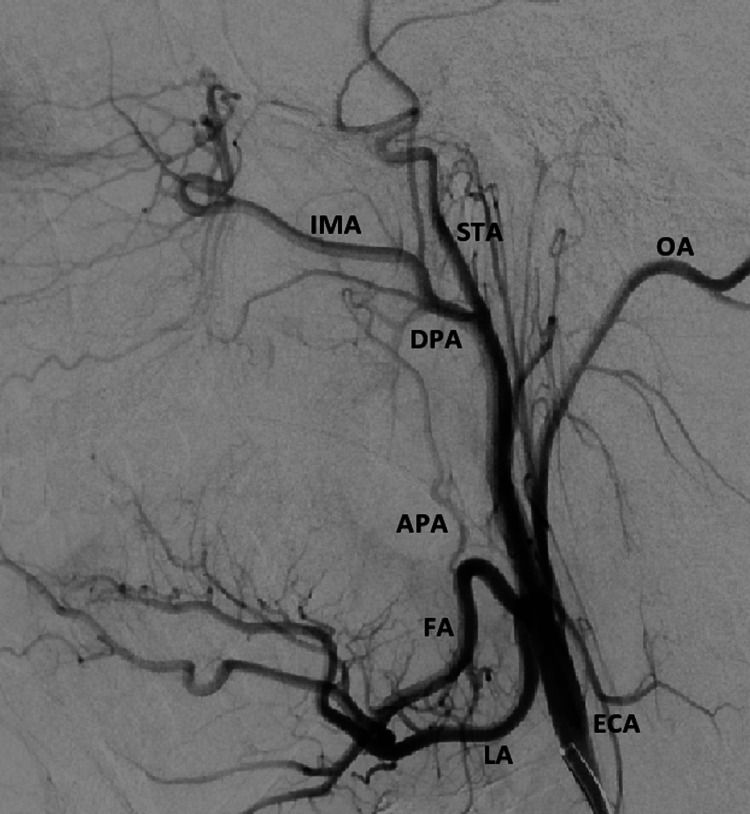
Pre-embolization digital subtraction angiography (lateral view) of the right external carotid artery showing various branches ECA: External carotid artery, LA: Lingual artery, FA: Facial artery, IMA: Internal maxillary artery, STA: Superficial temporal artery, OA: Occipital artery, APA: Ascending palatine artery, DPA: Descending palatine artery

**Figure 2:**
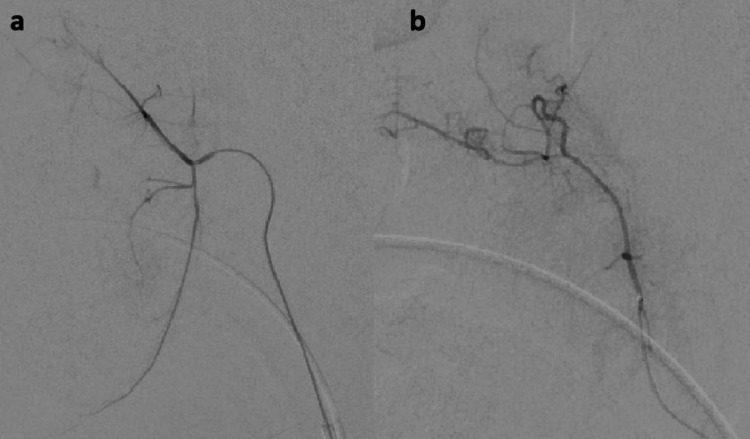
Selective digital subtraction angiography (lateral view) from the descending palatine artery (a) and the ascending palatine artery (b) did not reveal any obvious source of bleeding

**Figure 3:**
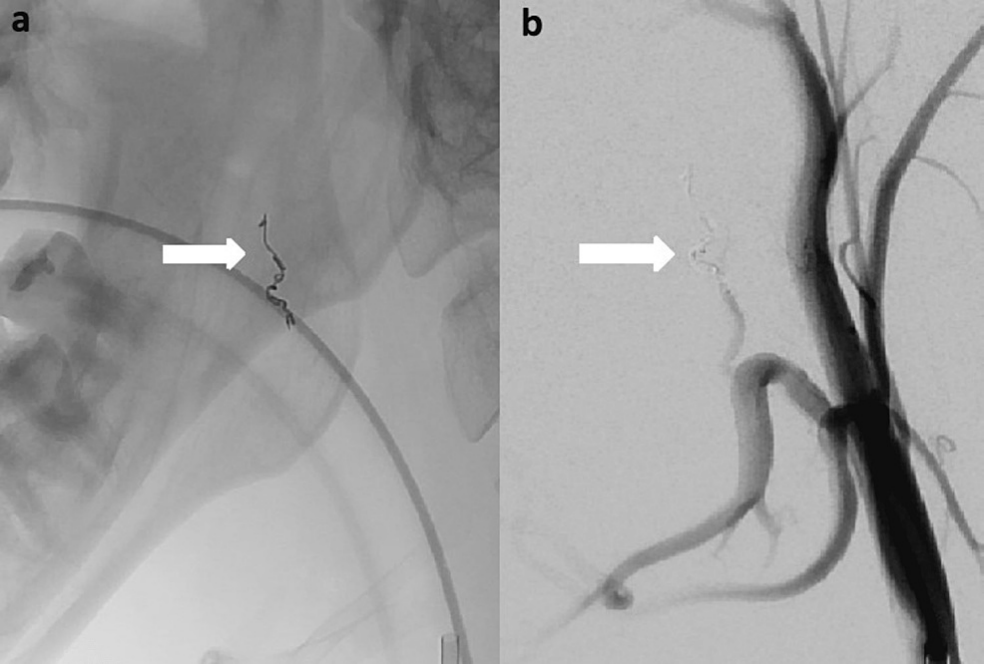
a) X-ray of the face (lateral view) showing microcoils in the ascending palatine artery. b) Post-embolization digital subtraction angiography (lateral view) of the ECA showing occlusion of the ascending palatine artery ECA: External carotid artery

The procedure was performed under general anesthesia. A 6 French 23 cm Arrow sheath (Teleflex, Morrisville, NC) was placed in the right common femoral artery. A 6 French 100 cm MPC guide catheter (Codman Neurovascular, Raynham, MA) was used for diagnostic angiography of the right external carotid artery. Figure 1 shows digital subtraction angiography (lateral view) of the right external carotid artery. Super-selective angiography of right ascending and descending palatine arteries was performed with an SL 10 microcatheter (Stryker Neurovascular, Fremont, CA) (Figure 2). No obvious source of bleeding was identified at this point. A dominant blood supply of the right tonsillar fossa was noted from the ascending palatine artery. After a discussion with the otolaryngologist, we decided to embolize the right ascending palatine artery, as it was the major arterial supply to the tonsillar fossa. Two orbit galaxy coils (2mm x 2cm, 2mm x 1.5cm) (Codman Neurovascular, Raynham, MA) were used to embolize the right ascending palatine artery (Figure 3a). Post-embolization external carotid artery angiography (Figure 3b) showed occlusion of the proximal right ascending palatine artery.

## Discussion

Tonsillectomy is among the most common surgical procedures in the United States, with more than 530,000 procedures performed annually in children younger than 15 years [1]. Recurrent throat infections and sleep-related breathing disorders from adenotonsillar hyperplasia are the common indications of tonsillectomy [1,2]. Palatine tonsils are highly vascular structures receiving blood supply from the branches of the facial, lingual, maxillary, and ascending pharyngeal arteries. Due to highly vascular anatomy, post-tonsillectomy hemorrhage remains the most common serious complication during and after surgery. The incidence of post-tonsillectomy hemorrhage is reported around 3% [1,2]. Post-tonsillectomy hemorrhage is classified into primary and secondary based on the timing of bleeding from the surgery. Primary post-tonsillectomy hemorrhage occurs within 24 hours post-surgery and is thought to be related to the surgical technique or a bleeding diathesis [3,4]. Secondary or delayed hemorrhage occurs beyond the first 24 hours postoperatively, most commonly five to 10 days after surgery when the slough separates from the granulating fossa [3,4]. Numerous factors, including older age, history of chronic tonsillitis, usage of nonsteroidal anti-inflammatory drugs, elevated postoperative mean arterial pressure, and excessive intraoperative blood loss, have been suggested to increase the risk of secondary hemorrhage [4].

Depending on the severity of post-tonsillectomy hemorrhage, initial management is usually observation or surgical intervention. Various surgical hemostasis techniques include the application of direct pressure to the bleeding site, topical hemostatic agent application, electrocautery, suturing of the tonsillar pillars, and surgical reexploration and ligation of bleeding vessels arising from the external carotid artery [5,6]. Success rates of surgical hemostasis after a first failed surgical procedure are low, about 50% for the second surgical procedure and 67% for the third [2]. Surgical hemostasis is challenging, for various reasons: (1) inability to visualize and locate the bleeding vessel in cases of profuse bleeding, (2) retraction of the bleeding vessel within the inflammatory pharyngeal tissue, and (3) pseudo-hemostasis achieved intraprocedurally due to reduced blood pressure linked to the hemorrhage or to general anesthesia [2].

Evidence for endovascular embolization of the branches of the external carotid artery as an effective treatment for severe post-tonsillectomy hemorrhage when attempts at surgical control have failed has been growing [2-5,7,8]. Endovascular embolization has several advantages, including: (1) the diagnostic angiographic assessment and subsequent intervention can be performed during the same procedure, (2) treatment can be targeted to one of the feeding arteries of the tonsillar fossa, as described in our case, (3) significantly shorter length of hospitalization, and (4) lower red blood cell transfusions during and post-procedure [2-5]. Nonetheless, endovascular embolization has its own risks. Potential risks of endovascular embolization include catheter-or wire-induced vasospasm, vessel perforation leading to extravasation of embolic material, ischemic injury to the mucosal surface and cranial nerves, inadvertent embolization of the internal carotid artery branches, and femoral artery injuries, including dissection, pseudoaneurysm, or rupture [4,5]. Various embolic materials, including coils, polyvinyl alcohol particles, and n-butyl-2-cyanoacrylate (NBCA) glue have been used for embolization [2-5,7,8].

Given the paucity of literature on endovascular embolization for severe post-tonsillectomy hemorrhage, we report our case to strengthen the evidence on feasibility, safety, and efficacy of endovascular embolization for severe surgically refractory post-tonsillectomy hemorrhage.

## Conclusions

Endovascular embolization of the branches of the external carotid artery is a safe and effective treatment for severe surgically refractory post-tonsillectomy hemorrhage. This treatment should be considered when standard surgical interventions have failed.
